# Experimental and Theoretical Studies of α-Linolenic Acid as Green Corrosion Inhibitor for Carbon Steel in 0.5 M Sulfuric Acid

**DOI:** 10.3390/molecules26206169

**Published:** 2021-10-13

**Authors:** I.A. Hermoso-Diaz, R. Lopez-Cecenes, J.P. Flores-De los Rios, L.L. Landeros-Martínez, E. Sarmiento-Bustos, J. Uruchurtu-Chavarin, J.G. Gonzalez-Rodriguez

**Affiliations:** 1 Department of Chemical Engineering and Applied Chemistry, University of Toronto, Toronto, ON M5S 3E5, Canada; alondra.hermosodiaz@utoronto.ca; 2 Chemical Science and Engineering Faculty, Morelos State Autonomous University, Cuernavaca 62209, Mexico; rlopez@uaem.mx; 3 Chemical Science Faculty, Chihuahua Autonomus University, Chihuahua 31125, Mexico; jpdelosrios@uach.mx; 4 Engineering Faculty, Chihuahua Autonomus University, Chihuahua 31125, Mexico; lilanderos@uach.mx; 5 Industrial Mechanical Division, Morelos State Emiliano Zapata Technological University, Emiliano Zapata 62565, Mexico; estelasarmiento@utez.edu.mx; 6 Research Centre in Engineering and Applied Sciences, Morelos State Autonomous University, Cuernavaca 62209, Mexico; juch25@uaem.mx

**Keywords:** carbon steel, green inhibitor, acid corrosion, quantum chemistry

## Abstract

A component of *Salvia hispanica*, α-linolenic acid, has been evaluated as a green corrosion inhibitor for 1018 carbon steel in 0.5 M sulfuric acid using weight loss tests, potentiodynamic polarization curves and electrochemical impedance spectroscopy (EIS) measurements. Theoretical calculations using Density Functional Theory (DFT) were used also. The results have shown that this compound is a good corrosion inhibitor, with an efficiency which increased with an increase in its concentration up to 600 ppm, but it decreased with a further increase in the concentration. α-linolenic acid formed protective corrosion products layer because it was chemically adsorbed onto the steel surface according to a Langmuir type of adsorption isotherms. Polarization curves have shown that α-linolenic acid is a good, mixed type of inhibitor with a predominant effect on the cathodic hydrogen evolution reactions. EIS measurements indicated a charge transfer-controlled corrosion process. DFT calculations indicated that α-linolenic acid was more efficient in an acidic environment than in a neutral one because has a high tendency to donate electrons and can be easily protonated. In addition to this, it had the highest E_HUMO_ value, the best chemical reactivity, the greatest tendency to transfer electrons and a greater facility of modifying its electronic configuration in the presence of carbon steel specimens according to its chemical hardness value.

## 1. Introduction

The study of corrosion of metals and alloys is a very important fact from both academic and industrial points of view due to big economical losses and many accidents it causes to a country [1,2]. There exist many processes in the industry where involved metals are in contact with aggressive solutions such as sulfuric (H_2_SO_4_), hydrochloric (HCl) and phosphoric acid (H_3_PO_4_), which produce degradation on them [2]. One of the most widely used forms to fight metals corrosion is the use of corrosion inhibitors [3]. For the particular case of iron and steel, there exist a high number of synthetic inhibitors [4,5,6]; however, they have the main drawback that they are toxic, harmful for the environment and humans and are very expensive. Thus, there is an increasing need to use more environmentally friendly corrosion inhibitors, such as those obtained from plant seeds, leaves, roots, etc., which are called “naturally occurring corrosion inhibitors” [7,8,9,10,11,12,13,14,15,16,17,18,19,20,21,22,23,24,25]. In the last decade, an extensive number of research works related to these kinds of substances have been published. For instance, *Pimenta dioica* was evaluated by Anupama et al. [26] as a green corrosion inhibitor for mild steel in HCl. For this, polarization curves, electrochemical impedance spectroscopy and theoretical computational calculations for the molecular components were used. In another work [27], modified lignin was evaluated as a green corrosion inhibitor for mild steel in HCl by using the same electrochemical and gravimetric techniques. The synergistic effect of several naturally occurring inhibitors, including polyaspartic acid, polyepoxysuccinic acid, polyamino polyether methylene phosphonate, sodium gluconate and Zn^2+^, were evaluated by Zhang et al. [28] for carbon steel in soft water. In a different study, Banana peels were evaluated as green corrosion inhibitors for mild steel in HCl. The authors found big differences between raw and ripe banana peel extracts [29]. Similarly, *Brassica oleracea* extract was evaluated as a naturally occurring inhibitor for X-52 pipeline steel in 0.5 M H_2_SO_4_ [30], finding an increase in the inhibitor efficiency with the inhibitor concentration and temperature up to certain values. A further increase in both the inhibitor concentration and testing temperature produced a decrease in the inhibitor efficiency. In a similar way, *Retama monosperma* [31], *Eleusine aegyptiaca* and *Croton rottleri* leaf extracts [32] have been evaluated as green corrosion inhibitors for mild steel in HCl.

In Mexico, since many centuries ago, *Salvia hispanica* L. has been a widely used seed for food and medicine [33,34] due to the fact that its fatty acids are highly unsaturated, containing mainly linoleic and α-linolenic acids, two polyunsaturated fatty acids, which cannot be produced in the human body, in addition to myrcetin, quercetin, kaempferol and caffeic acid, which act as potent antioxidants. Decreasing the level of cholesterol and triglyceride is one of the most important beneficial effects, as well as reducing blood pressure and the risk of heart disease. Additionally, some anti-inflammatory properties have been attributed to their omega-3-containing acids in *Salvia hispanica*, which make them beneficial for patients with rheumatoid arthritis and lupus [35,36]. *Salvia hispanica* extract has been previously evaluated as a green corrosion inhibitor for 1018 carbon steel in 0.5 M H_2_SO_4_ [37]. It was reported that *Salvia hispanica* inhibited both uniform and localized types of corrosion, with an efficiency that increased with increasing its concentration but decreased with increasing the testing temperature. The pitting potential increased and the passive current density decreased with increasing the inhibitor concentration. Thus, the goal of this paper is to evaluate the use of α-linolenic acid, one of the main components of *Salvia hispanica,* as a corrosion inhibitor for 1018 carbon steel in 0.5 M H_2_SO_4_ to know whether α-linolenic acid is mainly responsible for the corrosion inhibitive ability that *Salvia hispanica* possesses. In addition to this, to find a correlation between the corrosion inhibition properties and the inhibitor structure, the Density Functional Theory (DFT), a widely used technique [8,9,10,38,39,40,41], was used.

## 2. Experimental Procedure

### 2.1. Testing Material

Testing material includes 1018 carbon steel rods 6 mm in diameter containing 0.14% C, 0.90% Mn, 0.30% S, 0.030% P and balanced Fe. For weight loss tests, they were abraded up to 600-grade emery paper, washed and dried with hot air.

### 2.2. Testing Solution

**Figure 1:**
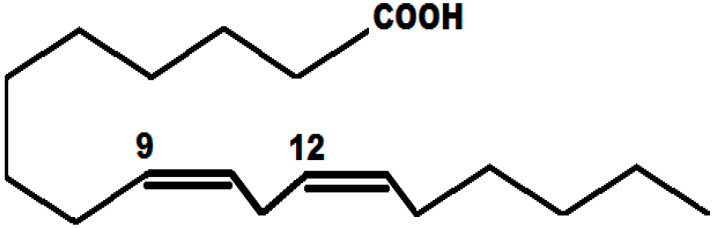
Chemical structure of α-linolenic acid.

The testing solution included 0.5 M H_2_SO_4_ prepared with analytical grade reagents and distilled water. α-linolenic acid was purchased from Sigma-Aldrich (Mexico City, Mexico) with a molecular structure, as shown in Figure 1. The concentrations used include 100, 200, 400, 600, 800 and 1000 ppm.

### 2.3. Weight Loss Measurements

For each inhibitor concentration, three specimens were used, and tests lasted 72 h at room temperature (25 °C), 40 and 60 °C. Specimens weight was taken before and after tests. Weight loss, Δm, was calculated as follows:

Δm = (m_2_ − m_1_)/A (1)

where m_2_ and m_1_ are the specimen’s weight before and after the tests, respectively, and A is the specimen area. On the other hand, inhibitor efficiency, I.E., was calculated as follows:

I.E. (%) = (Δm_2_ − Δm_1_)/Δm_2_ × 100 (2)

where Δm_2_ and Δm_1_ are the weight loss in the absence and presence of the inhibitor, respectively.

### 2.4. Electrochemical Techniques

#### 2.4.1. Potentiodynamic Polarization Curves

For electrochemical tests, coupons of carbon steel were encapsulated in a commercial resin, leaving an exposed area of 0.28 cm^2^. Employed electrochemical techniques include potentiodynamic polarization curves and electrochemical impedance spectroscopy (EIS). A standard three-electrode glass cell was used for these experiments, with a saturated calomel electrode (SCE) as the reference electrode and a graphite rode as the auxiliary electrode. Before starting the experiments, the free corrosion potential value, E_corr_, was measured until it reached a stable value, normally 30 min. For potentiodynamic polarization curves, steel was polarized from −1000 up to +2500 mV with respect to the E_corr_ value, at a scan rate of 1 mV/s. Inhibitor efficiency percent values, I.E., were obtained according to the next equation:

I.E. (%) = 100(Icorr_1_ − Icorr_2_)/Icorr_2_ (3)

where I_corr2_ and I_corr1_ are the corrosion current density values in the presence and absence of the inhibitor, respectively, which were calculated by using the Tafel extrapolation method. 

#### 2.4.2. Electrochemical Impedance Spectroscopy Studies

EIS measurements were carried out in a PC4 300 Gamry potentiostat (Queretaro, Mexico) by applying a signal of ±10 mV around the E_corr_ value at the interval frequency between 10 KHz and 0.5 Hz, obtaining 50 points per decade. Inhibitor efficiency values were calculated using

I.E. (100%) = 100 (R_ct2_ − R_ct1_)/R_ct2_ (4)

where R_ct2_ and R_ct1_ are the charge transfer resistance values with and without the addition of the inhibitor, respectively.

### 2.5. Computational Studies

In order to try to find a correlation between the inhibitory properties and the electronic properties of the α-linolenic acid, some quantum chemical calculations were performed. Quantum chemical calculation is the most appropriate method to investigate adsorption and inhibition mechanism. In order to construct a composite index of an inhibitor molecule, it may be important to focus on parameters that directly influence the electronic interaction of the inhibitor molecules with the metal surface. For this, the Density Functional Theory (DFT) with B3LYP Beck’s three-parameter exchange functional along with Yang and Parr non-local correlation functional (B3LYP) and 6–311G^+^ basis [42,43] was used. The energies of the highest occupied molecular orbital (E_HOMO_) and lowest unoccupied molecular orbital (E_LUMO_), as well as the dipolar moment, μ, are very important quantum chemical parameters. A computational study was performed in the presence of water as the solvent, using the IEFCPCM (integral equation formalism PCM) method coupled to UAKS radii in the PCM (polarizable Continuum Model) [44,45].

Within the framework of the Density Functional Theory, the chemical potential is defined as the negative of the electronegativity.

X = −(∂E/∂N)(5)

Whereas the hardness (η) is defined as:

η = 1/2 (∂^2^E/∂N^2^) (6)

where E is the electronic energy and N is the number of electrons.

I and A are related in turn to HOMO and LUMO, using equations:

I = −HOMO(7)

A = −LUMO(8)

These quantities are related to the electron affinity (A) and ionization potential (I) using the following equations:

X = (I + A)/2, X = −(LUMO + HOMO)/2(9)

η = (I − A)/2, X = −(LUMO − HOMO)/2(10)

The global electrophilicity index (*ω*) is:

*ω* = μ^2^/2η (11)

The Fukui function (FF), which measures reactivity in a local sense, using a scheme of finite difference approximations. This procedure condenses the values around each atomic site into a single value that characterizes the atom in the molecule. With this approximation, the condensed Fukui becomes:

*f*^+^_k_ = q_k_(N + 1) − q_k_(N) (for nucleophilic attack) (12)

*f*^−^_k_ = q_k_(N) − q_k_(N − 1) (for electrophilic attack) (13)

where q_k_(N + 1), q_k_(N) and q_k_(N − 1) are charge values of atom k for cation, neutral and anion, respectively [46].

Similar to FF for a nucleophilic and electrophilic attack, the local softness and local philicity were used to provide more information on the reactivity of atoms in the α-linolenic acid molecule.

The local softness [47] and local philicity [48] are given by

*S*_k_^α^ = *S f*_k_ ^α^ (14)

*ω*_k_^α^ = *ω f*_k_ ^α^ (15)

where α = +,− represent nucleophilic and electrophilic attacks respectively.

On the other hand, the dual local descriptors, such as dual Fukui Δ*f*_k_, is defined as the difference between the nucleophilic and electrophilic Fukui functions [49]. The dual local softness Δ*S*_k_ (difference between the nucleophilic and electrophilic local softness) and the philicity Δ*ω*_k_ (difference between the nucleophilic and electrophilic local philicity) are more accurate and consistent tools than the aforementioned local reactivity indices [50]. Δ*f*_k_, Δ*S*_k_ and Δ*ω*_K_ are given by

Δ*f*_k_ = *f*_k_^+^ − *f*_k_^−^ (16)

Δ*S*_k =_ *S*_k_^+^ − *S*_k_^−^ (17)

Δ*ω*_k =_ *ω*_k_^+^ − *ω*_k_^−^ (18)

## 3. Results and Discussion

### 3.1. Weight Loss

**Figure 2:**
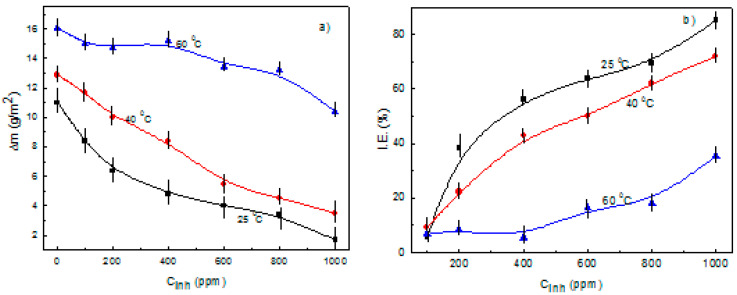
Effect of α-linolenic acid concentration on the (**a**) weight loss and (**b**) inhibitor efficiency for carbon steel in 0.5 M H_2_SO_4_ at different temperatures.

**Figure 3:**
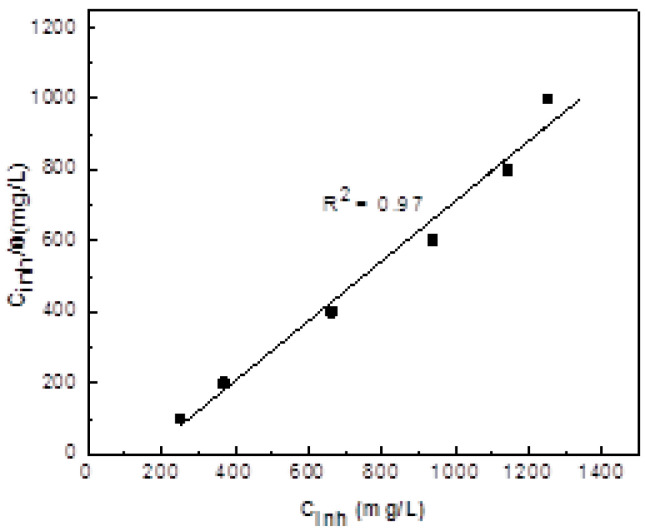
Langmuir adsorption isotherm for carbon steel in 0.5 M H_2_SO_4_ in the presence of α-linolenic acid.

The change in the weight loss and inhibitor efficiency with the inhibitor concentration at the different testing temperatures is given in Figure 2. This figure shows that the weight loss decreases with an increase in the inhibitor concentration, but it increases with an increase in the temperature. Additionally, inhibitor efficiency increases with an increase in the inhibitor concentration, but it decreases when the testing temperature increases. Similar results were obtained by Hermoso-Diaz et al. [37], who worked with *Salvia hispanica* extract but obtained a highest inhibitor efficiency value of 91% with the addition of 1000 ppm, whereas, in this research work, the highest inhibitor efficiency was 85%, but with less of the inhibitor, it became 600 ppm. This decrease in the metal weight loss is due to the adsorption of α-linolenic acid onto the steel surface with the formation of a layer of protective corrosion products, and, in order to elucidate on the way that the inhibitor is adsorbed, different adsorption isotherms, including Langmuir, Temkin and Frumkin. As can be seen from Figure 3, the best fitting was obtained with the Langmuir type of adsorption isotherm [51], which is given by:

(19) $$\frac{C_{\mathrm{inh}}}{\theta }=\frac{1}{K_{\mathrm{ads}}}+C_{\mathrm{inh}}$$

where the inhibitor concentration is represented by C_inh_, K_ads_ is the adsorption constant = 3.12 × 10^−2^ L/mg, and θ represents the surface coverage, obtained by dividing the inhibitor efficiency by 100.

There exists a relationship between the adsorption constant, K_ads_, and the standard adsorption free energy,$\;\Delta G_{\mathrm{ads}}^{0}$, which is:

(20) $$\Delta G_{\mathrm{ads}}^{0}=-\mathrm{RT}\;\mathrm{In}\;\left(10^{6}{\;K}_{\mathrm{ads}}\right)$$

where R is the universal gas constant, and T is the absolute temperature. From the intercepts of the straight lines C_inh_/θ—Y axis, and by knowing that the concentration of water in the solution is 106 mg/L, the value obtained for $\Delta G_{\mathrm{ads}}^{0}$ is −43.34 kJ mol^−1^. Generally, for values of $\Delta G_{\mathrm{ads}}^{0}\;$ around −20 kJ mol^−1^ or less negative, the type of adsorption is regarded as physisorption; those around −40 kJ mol^−1^ or more negative are associated with chemisorption [51]; therefore, a value of −43.34 kJ mol^−1^ indicates a chemical adsorption [51]. The negative value for $\Delta G_{\mathrm{ads}}^{0}\;$ indicates that the adsorption process is spontaneous.

**Figure 4:**
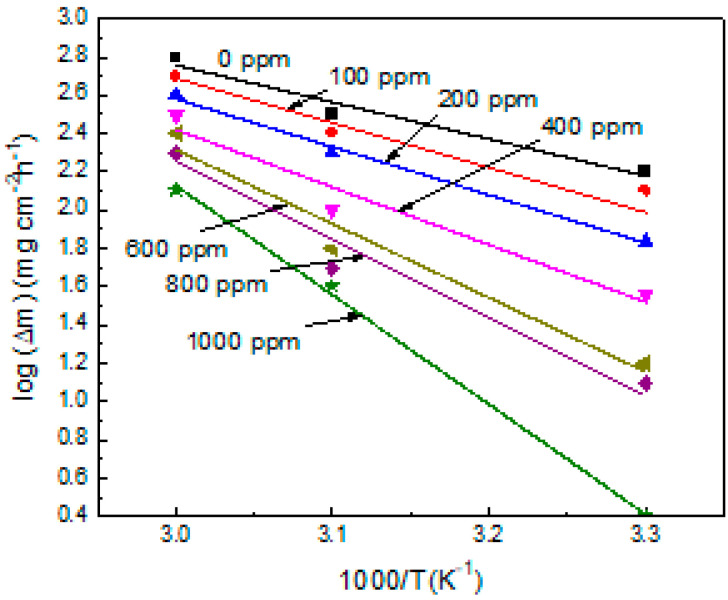
Arrhenius E_a_ activation energy plots for carbon steel in 0.5 M H_2_SO_4_ containing different concentrations of α-linolenic acid.

Activation energy, E_a_, for carbon steel in the absence and presence of α-linolenic acid was calculated using the Arrhenius type of equation:

log (∆m) = −E_a_/2.3RT + A (21)

where A is the Arrhenius pre-exponential factor. By plotting the weight loss results at the different inhibitor concentrations as a function of −1/2.3 RT, as shown in Figure 4, the different values of activation energy can be obtained, and these results are given in Table 1. Table 1 shows that the activation energy values are larger in the presence of the inhibitor, which designates that the adsorption of inhibitor is mainly the physical (electrostatic) adsorption up to a concentration of 100 ppm, while the chemical bonding would arise between the inhibitor molecules and the steel surface at inhibitor concentrations higher than 100 ppm [52,53].

### 3.2. Polarization Curves

**Figure 5:**
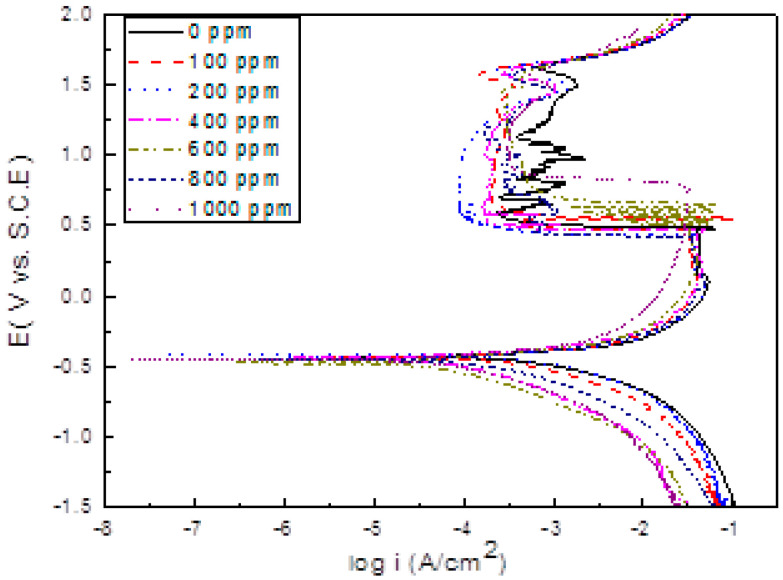
Effect of α-linolenic acid concentration in the polarization curves for 1018 carbon steel in 0.5 M H_2_SO_4_.

The effect of α-linolenic acid concentration in the polarization curves for 1018 carbon steel in 0.5 M H_2_SO_4_ is shown in Figure 5. It can be seen that in all cases, curves display an active-passive behavior, where the E_corr_ value practically was unaffected by the addition of α-linolenic acid, as can be seen in Table 2. The free corrosion potential for the uninhibited solution was −430 mV, and it remained around this value with the addition of the different inhibitor concentrations up to 400 ppm, but when 600 ppm or higher inhibitor concentrations were added, the E_corr_ reached more active values than those obtained in the absence of inhibitor, between −455 and −472 mV. The corrosion current density value, I_corr_, decreased, and the polarization resistance value increased with the addition of the inhibitor up to 600 ppm, but they followed the opposite behavior with a further increase in the inhibitor concentration. The decrease in the I_corr_ value with an increase in the inhibitor concentration is due to the adsorption of the α-linolenic acid on the steel surface [29,30,31,32].

Inhibitor efficiency also increased when increasing the inhibitor concentration, reaching its highest value when 600 ppm of α-linolenic acid was added, and it decreased with a further increase in the α-linolenic acid concentration. Hermoso-Diaz et al. [37] obtained a maximum inhibitor efficiency of 99% with the addition of 1000 ppm of *Salvia hispanica*, whereas, in the present work, the maximum inhibitor efficiency was 97% but with the addition of less of the inhibitor, i.e., 600 ppm. As established in [37], α-linolenic acid is one of the main compounds found in *Salvia hispanica* extract together with linoleic acid and quercetin. Thus, the higher efficiency obtained with the *Salvia hispanica* extract must be by a kind of synergistic effect of these components and not only to the effect of α-linolenic acid. Thus, one of the goals of the present work, which was to determine whether or not the obtained inhibitor efficiency for *Salvia hispanica* extract was due to the presence of α-linolenic acid within its structure has been reached, and it has been found that α-linolenic acid is partially responsible for this inhibition efficiency. However, we must take into account that α-linolenic acid is an expensive reagent and its inhibition efficiency is lower than that obtained with *Salvia hispanica* extract.

The passive current density value, I_pas_, was decreased in the same fashion, reaching its lowest value with the addition of 600 ppm, and it increased once again when the inhibitor concentration increased further. The pitting potential value, E_pit_, was marginally affected by the addition of the inhibitor, fluctuating between 1580–1640 mV, obtaining its highest value in the uninhibited solution. Finally, the cathodic Tafel slope was affected to a greater extent than the anodic Tafel slope by the addition of the inhibitor, indicating that the α-linolenic acid acted as a mixed type of inhibitor, affecting both anodic and, mainly, the cathodic reaction, such as hydrogen evolution reaction, maybe by a blocking effect [54,55] since at low pH value, H^+^ reduction is the dominant cathodic reaction because of the high H^+^ concentration [56].

### 3.3. EIS Measurements

**Figure 6:**
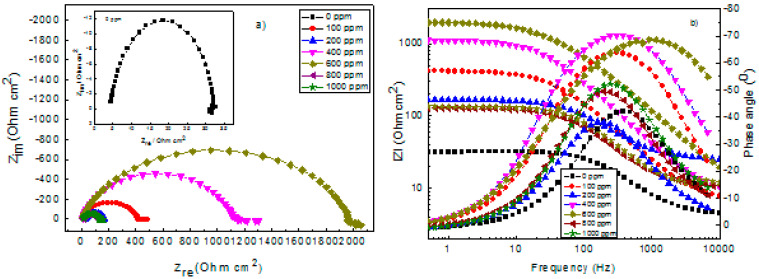
Effect of α-linolenic acid concentration in the (**a**) Nyquist and (**b**) Bode diagrams for 1018 carbon steel in 0.5 M H_2_SO_4_.

EIS measurements in the Nyquist and Bode formats for 1018 carbon steel with and without α-linolenic acid are shown in Figure 6. Nyquist data, Figure 6a, display a single, depressed, capacitive-like semicircle at all the frequency values with its center at the real axis, indicating that the corrosion process is under charge transfer control. The fact that it is not a pure semicircle is due to the surface roughness during the corrosion process. When the inhibitor was added, the data still display a capacitive semicircle at high- and intermediate-frequency values followed by some kind of elongations, where the imaginary impedance remains constant, but the real part increases due to the accumulation of a layer of corrosion products on the steel surface [54]. The semicircle diameter increases as the inhibitor concentration increases, reaching its highest value at 600 ppm, decreasing its value with a further increase in the inhibitor concentration. On the other hand, the Bode diagrams, Figure 6b, show that the impedance increases with increasing the α-linolenic acid concentration, reaching its highest value in a concentration of 600 ppm, and it decreases when the inhibitor concentration is further increased. The Bode diagrams in the angle phase format, Figure 6b, show only one peak around 500 Hz for the uninhibited solution, which indicates the absence of any protective layer in this case. However, when α-linolenic acid is added into the solution, the phase angle starts to increase with the frequency, and it remains constant in a relatively wide range of frequency, especially with the addition of 400 or 600 ppm of the inhibitor. The presence of α-linolenic acid molecules at the interface consequently brings an increase in the peak heights. All spectra indicate the presence of a time constant only. The fact that the angle phase remains constant in a wide frequency interval indicates the formation of a protective layer formed by the inhibitor and Fe^2+^ ions on the steel surface, forming metal–inhibitor complexes. This frequency interval where the angle phase remains constant decreases for inhibitor concentrations higher than 600 ppm, indicating that the formed protective layer loses its protectiveness, bringing an increase in the corrosion rate.

**Figure 7:**
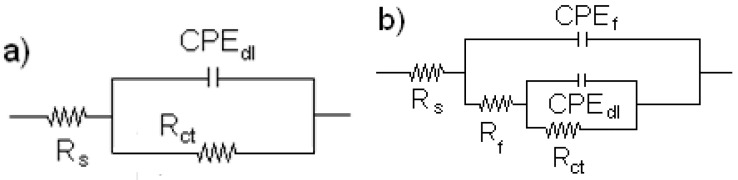
Equivalent electric circuits used to simulate EIS data for carbon steel in 0.5 M H_2_SO_4_ in the (**a**) absence and (**b**) presence of α-linolenic acid.

Equivalent electric circuits, such as those shown in Figure 7, are generally used to describe the process occurring at the metal–solution interface. In this figure, the solution resistance is represented by R_s_, the charge transfer resistance by R_ct_, the double-layer capacitance by C_dl_, the film formed by the corrosion products resistance R_f_ and C_f_ its capacitance. However, to take into account dispersion effects due to surface heterogeneities such as surface roughness due to its dissolution, ideal capacitances are replaced by constant phase elements, CPE. Table 3 summarizes the electrochemical parameters obtained from the fitting by using electric circuits shown in Figure 7. From Table 3, it can be seen that the R_ct_ value increases as the inhibitor concentration increases, reaching its highest value with the addition of 600 ppm, which is due to the adsorption of α-linolenic acid to form a protective, passive layer, as shown by the polarization curves in Figure 5. A further increase in the α-linolenic acid concentration brings a decrease in the R_ct_ value. The CPE_dl_ value, on the other side, decreases with an increase in the inhibitor concentration, reaching its lowest value at 600 ppm, increasing with a further increase in the inhibitor concentration. This decrease in the CPE_dl_ value is attributed to the replacement of the adsorbed water molecules at the metal surface by the inhibitor molecules [57] and forming a protective layer. The inhibitor efficiency increases with the α-linolenic acid concentration, reaching its maximum value when 600 ppm of the inhibitor is added to the electrolyte, and it decreases with a further increase in the α-linolenic acid concentration similar to the results given by polarization curves in Figure 5.

It is generally accepted that the first step during the adsorption of an organic inhibitor on a metal surface usually involves the replacement of water molecules absorbed on the metal surface:

Inh_sol_ + xH_2_O_ads_ → Inh_ads_ + xH_2_O_sol_ (22)

The inhibitor may then combine with freshly generated Fe^2+^ ions on the steel surface, forming metal inhibitor complexes [58,59]:

Fe → Fe^2+^ + 2e (23)

Fe^2+^ + Inh_ads_ → [Fe-Inh]_ads_^2+^ (24)

The resulting complex, depending on its relative solubility, can either inhibit or catalyze further metal dissolution. At low concentrations, the amount of α-linolenic acid is not enough to form a compact complex with the metal ions, so the resulting adsorbed intermediate will be readily soluble in the acidic environment. However, at relatively higher inhibitor concentrations, more α-linolenic acid molecules become available for the complex formation, which subsequently diminishes the solubility of the surface layer, leading to improve the inhibition of metal corrosion. Thus, so far, it has been shown that the increase in the impedance modulus is due to the protective character of the α-linolenic acid.

### 3.4. Theoretical Calculations

#### Neutral Molecule

**Figure 8:**
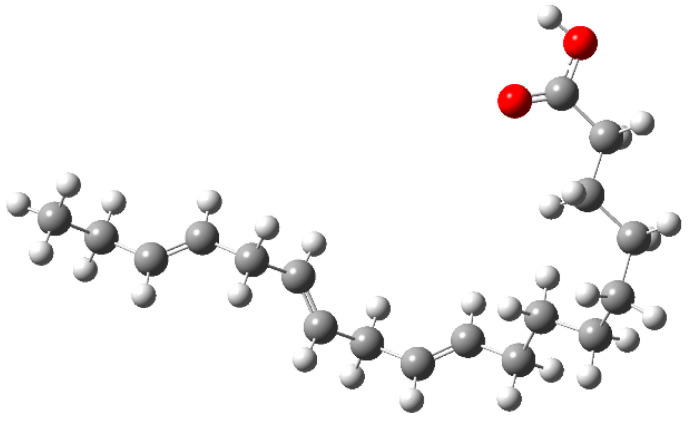
Optimized structure of the α-linoleic acid in the aqueous phase.

**Figure 9:**
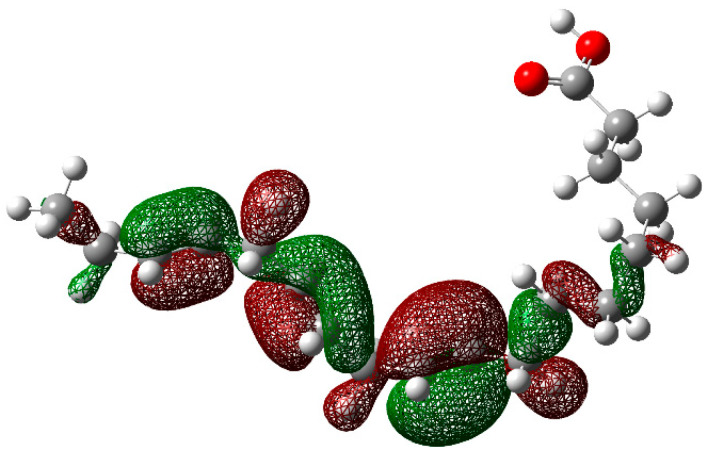
The highest occupied molecular orbital (HOMO) of α-linoleic acid in the aqueous phase.

**Figure 10:**
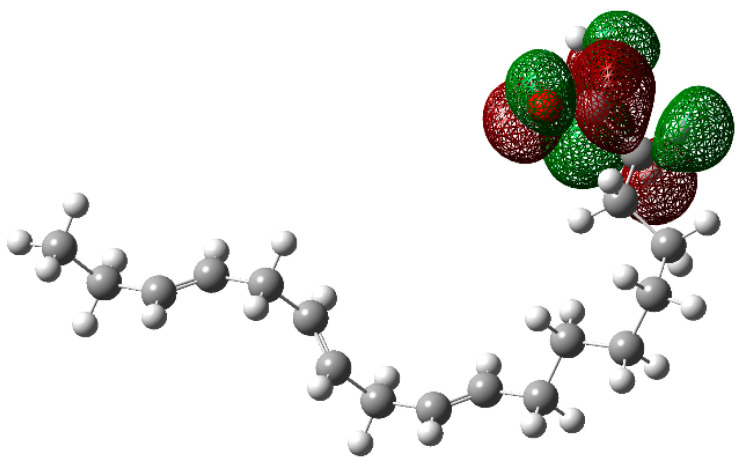
The lowest unoccupied molecular orbital (LUMO) of α-linoleic acid in the aqueous phase.

**Figure 11:**
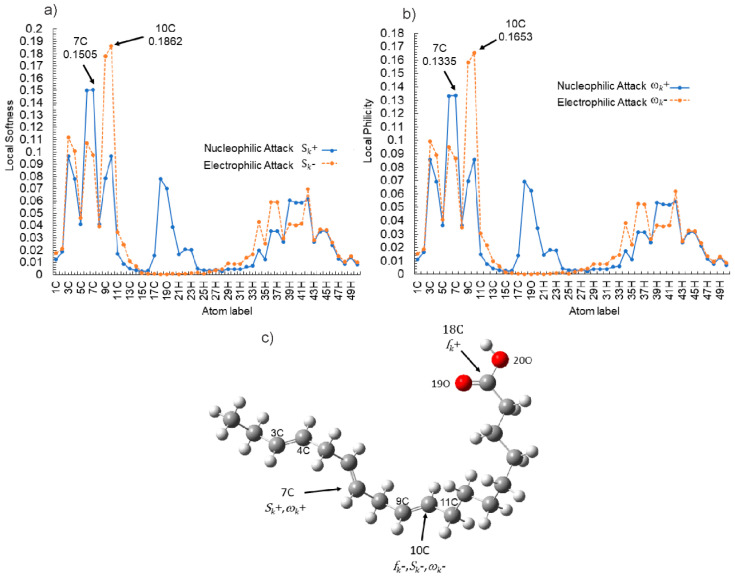
Graphical representation of the local dual descriptors nucleophilic and electrophilic attack (**a**) local softness *S*_k_^+^, *S*_k_^−^, (**b**) local philicity *ω*_k_^+^,*ω*_k_^−^ and (**c**) nucleophilic and electrophilic attack on hetero-atoms for the neutral α-linolenic acid using the Fukui function, local softness and local philicity.

In order to have a better understanding of the corrosion mechanism, some quantum chemical calculations to study the correlation between the molecular structure of this organic compound and its inhibition effect have been performed. Chemical parameters were discussed both in the aqueous phase. The optimized structure in its neutral state is shown in Figure 8. The molecule reactivity was investigated via analysis of the frontier molecular orbital. Electronic parameters are listed in Table 4. E_LUMO_ indicates the propensity of the molecule to accept electrons. The lower the E_LUMO_ value is, the greater is the ability of that molecule to accept electrons. The higher the E_HOMO_ is, the greater is the ability of that molecule to donate electrons [60]. The gap (ΔE) is an important parameter that indicates the reactivity tendency of organic molecules toward the metal surface. As ΔE decreases, the reactivity of the molecule increases, leading to an increase in its adsorption onto a metal surface. Normally, the lowest value of hardness is expected to have the highest inhibition efficiency. The high values of the dipolar moment (μ) favor the accumulation of the inhibitor on the surface. The electrophilicity index, on the other hand, denotes the electron-accepting capability of a molecule [61]. HOMO is often associated with electron-donating species. As can be seen in Figure 9, the HOMO is distributed over the entire chain of carbon atoms, and consequently, the calculated values of the Hirsfeld charges (Table 5) on hetero-atoms indicate that carbon atom number 10 is the prominent site for the electrophilic adsorption. Likewise, LUMO indicates its ability to accept electrons that are located in the carboxyl functional group. The orbitals map is shown in Figure 10. On the other hand, Table 5 shows that some of the results obtained of the condensed Fukui, where the nucleophilic attack (*f*_K_^+^) value is in the atom 18C, whereas electrophilic attack (*f*_K_^−^) is located in the atom 10C, Figure 11a, which is in agreement with the distribution of electronic density in HOMO and LUMO, respectively. Consequently, the local softness for electrophilic (*S*_k_^−^) and nucleophilic attacks (*S*_k_^+^), as well as the local philicity for electrophilic (*ω*_k_^−^) and nucleophilic attacks (*ω*_k_^+^), have been calculated for each atom in the molecule. According to these results, the highest value of *S*_k_^+^ is for atom 7C (0.1505) and *S*_k_^−^ is for atom 10C (0.1862) and belongs to the double-bound C=C in the same way the highest *ω*_k_^+^ and ω_k_^−^ values are 0.1335 in 7C atom and 0.1653 in 10C atom, respectively. The optimized structure α-linolenic acid contains an active center for protonation. According to *f*_K_^−^, *S*_k_^−^ and *ω*_k_^−^, the atom most susceptible to an electrophilic attack is 10C in all cases, being the most prominent site for electrophilic adsorption. While the prominent site for adsorption (nucleophilic) according to *f*_k_^+^ is 18C atom, and in *S*_k_^+^, *ω*_k_^+^ is 7C atom. The graphic of the local softness and philicity for the electrophilic attack (*S*_k_^−^, *ω*_k_^−^) and nucleophilic attack (*S*_k_^+^, *ω*_k_^+^) of α-linolenic acid is presented in Figure 11. 

**Figure 12:**
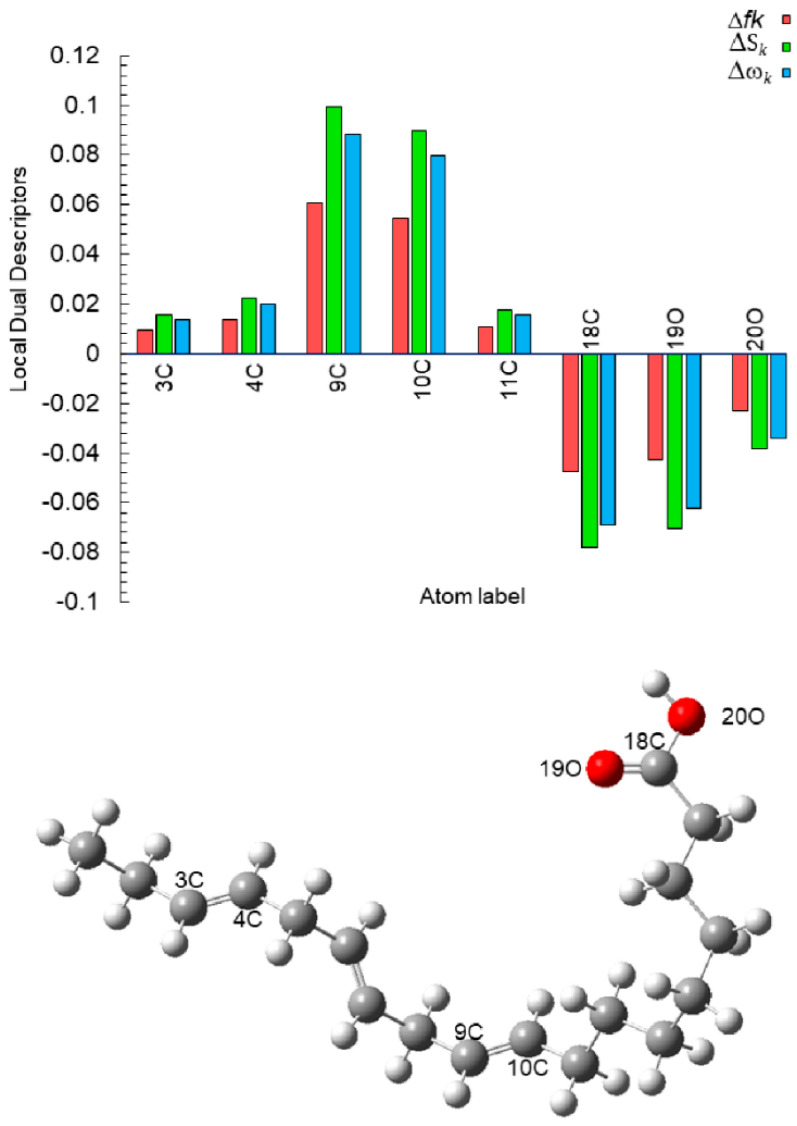
Graphical representation of the dual descriptors (Δ*f*_k_, Δ*S*_k_ and Δ*ω*_k_) for the most active sites of neutral α-linoleic acid molecule.

The results of the dual Fukui function (Δ*f*_k_), the dual local softness (Δ*S*_k_), and the dual local philicity (Δ*ω*_k_) are summarized in Table 5. The most representative active sites of de descriptors (Δ*f*_k_, Δ*S*_k_ and Δ*ω*_k_) are shown in Figure 12. According to Hsissou et al., the dual descriptors with values lower than zero have the ability to accept electrons from the metal, while those with values higher than zero suggest electrophilic centers [50]. The most active sites for electron-accepting centers for neutral α-linoleic acid decreases in the following order: 18C > 19C > 20C, whereas, in the active sites donation, the decreasing order is: 9C > 10C > 4C > 3C > 11C. 

**Figure 13:**
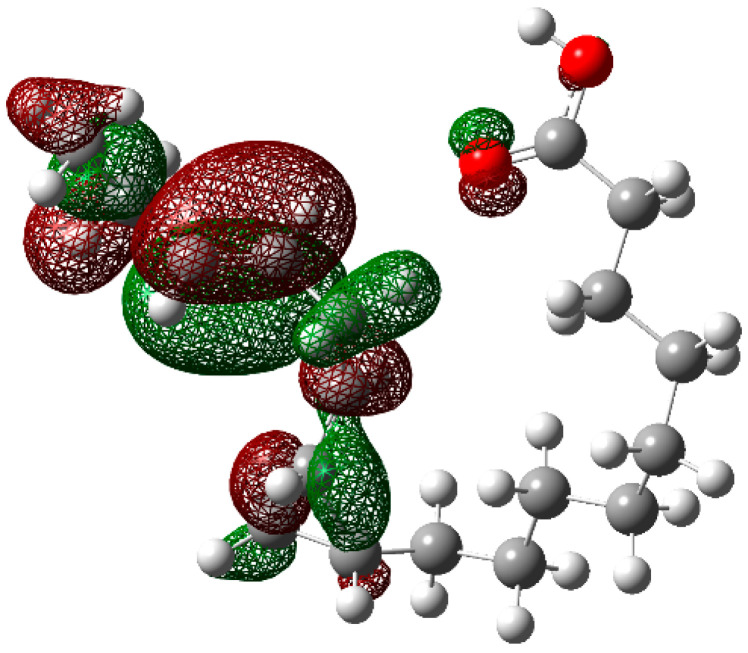
The highest occupied molecular orbital (HOMO) for the protonated α-linoleic acid in the aqueous phase.

**Figure 14:**
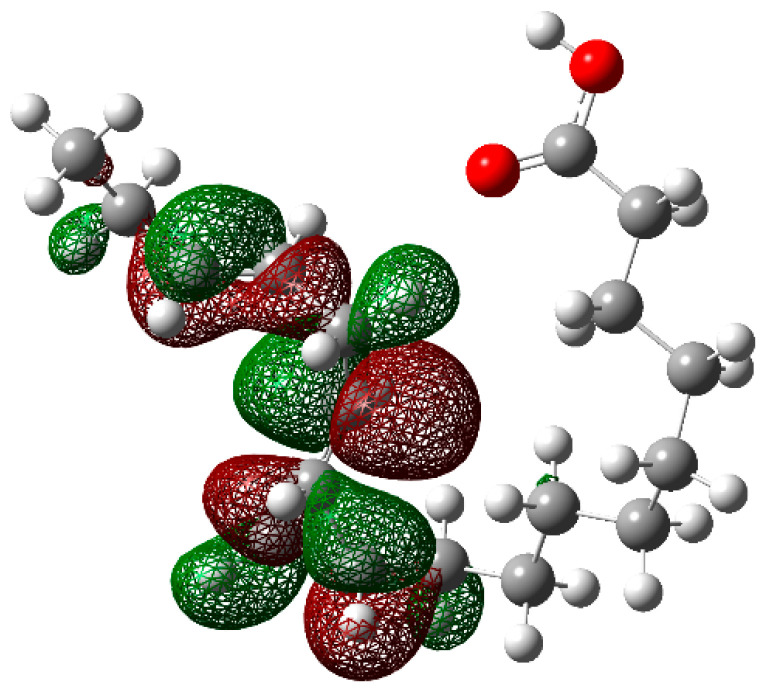
The lowest unoccupied molecular orbital (LUMO) for the protonated α-linoleic acid in the aqueous phase.

**Figure 15:**
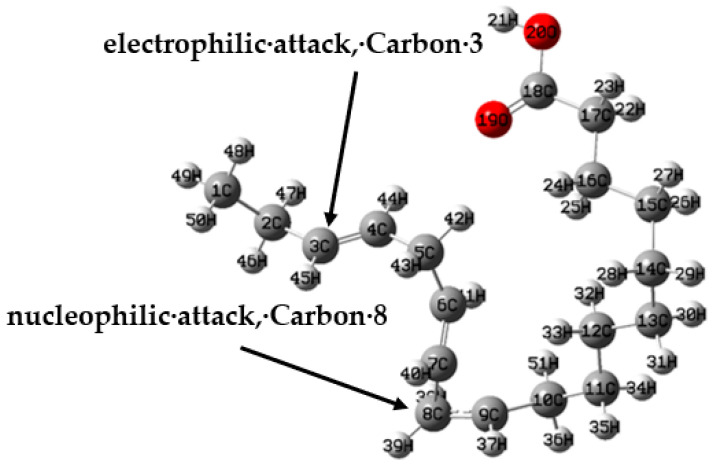
The nucleophilic and electrophilic attack in the protonated α-linoleic acid molecule using the Fukui function.

**Figure 16:**
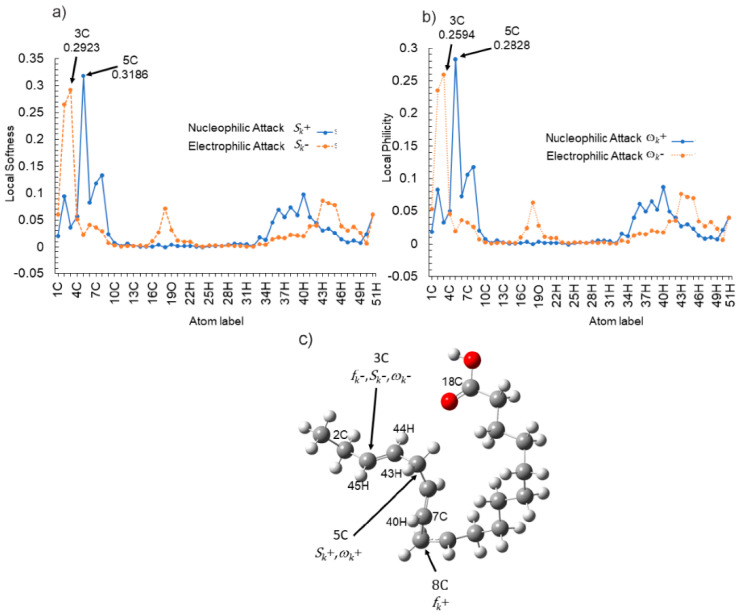
Graphical representation of the local dual descriptors nucleophilic attack and electrophilic attack (**a**) local softness *S*_k_^+^, *S*_k_^−^, (**b**) local philicity *ω*_k_^+^, *ω*_k_^−^ and (**c**) nucleophilic and electrophilic attack on hetero-atoms for the protonated α-linolenic acid using the Fukui function, local softness and local philicity.

The presence of the alkene functional group (C=C) in the structure of α-linolenic acid provides a high tendency towards its protonation in an aqueous acidic medium. Therefore, it is important to analyze the structures in their protonated form [61]. According to local descriptors, Figure 11, the most susceptible atom to an electrophilic attack (*f*_k_^−^, *S*_k_^−^, *ω*_k_^−^), on the α-linolenic acid is the atom 10C, which corresponds to a C=C bond. Because protonation is the addition of a proton (H^+^) to an atom, molecule, or ion, it was determined that atom 10C will be the site to be protonated [62]. Electronic parameters of the protonated inhibitor molecule and its optimized structure can be seen in Table 6 and Figure 13, respectively. A comparison of the quantum chemical calculations for neutral and protonated molecules indicates that there is a clear correlation. E_LUMO_ indicates the propensity of the molecule to accept electrons. The lower E_LUMO_ is, the greater is the ability of that molecule to accept electrons. However, active sites for donating or accepting electrons changed with respect to the neutral molecule. The electron clouds in both HOMO and LUMO occupy similar areas, as can be seen in Figure 14 and Figure 15, respectively. Some values with the Fukui functions, local softness and local philicity are shown in Table 7. The prominent site for the electrophilic adsorption according to *f*_k_^−^, *S*_k_^−^, *ω*_k_^−^ is in atom 3C; likewise, the prominent site for the adsorption (nucleophilic) in *f*_k_^+^ is atom 8C, and in *S*_k_^+^, *ω*_k_^+^ is atom 5C (See Figure 16).

**Figure 17:**
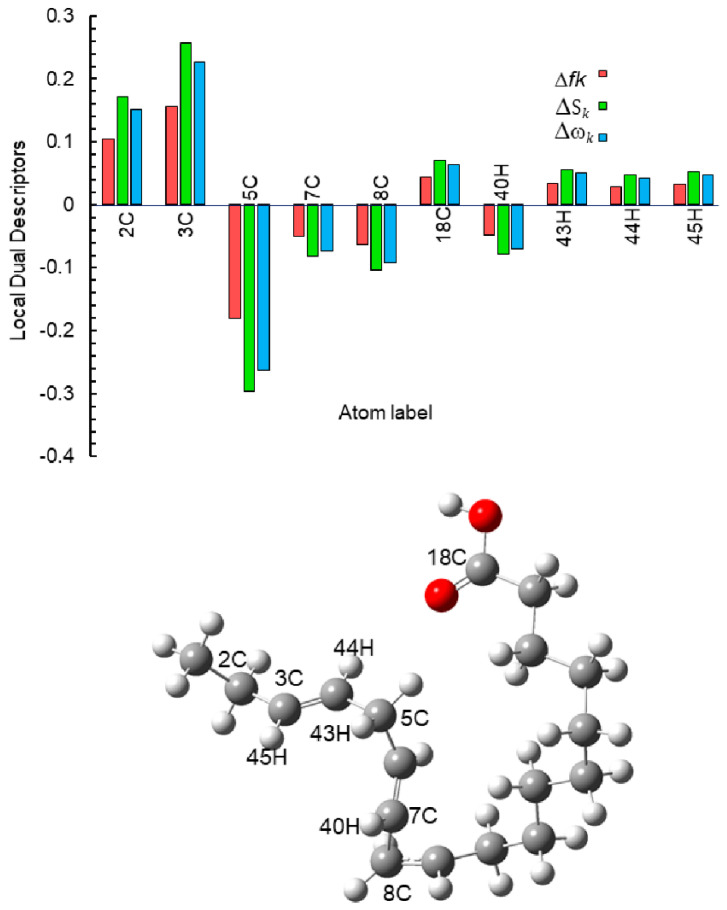
Graphical representation of the dual descriptors (Δ*f*_k_, Δ*S*_k_ and Δ*ω*_k_) for the most active sites of the protonated α-linoleic acid molecule.

The values of the dual Fukui function (Δ*f*_k_), the dual local softness (Δ*S*_k_) and the dual local philicity (Δ*ω*_k_) are shown in Table 7. The active sites for electron-accepting from metal centers in the protonated linoleic acid decreases in the following order: 5C > 8C > 7C > 40H, and the active sites for electron-donating from metal centers in decreasing order is: 3C > 2C > 18C > 43HC > 45H > 44H. See Figure 17.

In summary, according to Guo et al. [63], molecules that have back-donation at their carbon atoms are those where the electron density moves along the structure from HOMO to LUMO. According to these results, one could conclude that neutral α-linoleic acid will have some active centers (nucleophilic and electrophilic) to interact with the steel surface. In contrast to protonated α-linoleic acid, the electron density in both HOMO and LUMO is in the same part of the structure where *f*_K_^α^, *S*_k_^α^ and *ω*_k_^α^ allowed us to distinguish the atoms susceptible to both electrophilic and nucleophilic attack in the steel surface. Additionally, the local dual descriptors allow us to identify the active sites for electron-accepting and electron-donating in neutral and protonated states.

### 3.5. Corrosion Inhibition Mechanism

**Figure 18:**
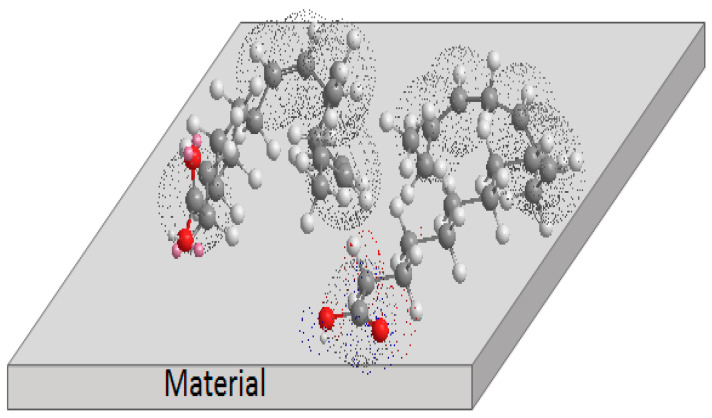
Adsorption process of α-linolenic acid on steel surface.

In order to try to explain the way that α-linolenic acid is adsorbed onto 1018 carbon steel and inhibits its dissolution, a scheme shown in Figure 18 will be used.

Based on the chemical structure for α-linolenic acid given in Figure 1, it can be seen that it contains a hydrophilic and a hydrophobic part within its structure. As it is known, when an H^+^ atom is attached to a C in a ring contained in an inhibitor, it improves its inhibition efficiency since it could form -CHO or -COOH groups [64]. Based on the latter, α-linolenic acid has a carboxyl group -COOH in its structure that corresponds to the hydrophilic part. The hydrophobic side corresponds to the carbon chain in the α-linolenic acid, which, as was explained, in the presence of H^+^, could result in -CHO or -COOH groups.

The carboxylic group is adsorbed on the metal surface to occupy one adsorption site. This adsorption is preferable by an electronic interaction between the unshared electrons pairs from the heteroatoms or the π orbitals bond from the molecule, with the d orbitals on the metallic surface. The alkyl chain is adsorbed on the surface of the material through the π orbitals of the unsaturation. 

## 4. Conclusions

This research has shown that α-linolenic acid turned out to be an efficient corrosion inhibitor for carbon steel in 0.5 M H_2_SO_4_, with its efficiency increasing with an increase in its concentration up to 600 ppm, but it decreases with a further increase in its concentration. Polarization curves have shown that α-linolenic acid is a good, mixed type of inhibitor with a stronger effect on the cathodic hydrogen evolution electrochemical reactions. This is because α-linolenic acid is chemically adsorbed onto the steel surface according to a Langmuir adsorption isotherm to form a layer of protective corrosion products. It decreased not only the corrosion current density but also the passivation current density value. EIS measurements indicated that the corrosion process was under charge transfer control. DFT calculations indicated a high tendency of α-linolenic acid to donate electrons and that it can be easily protonated in its O atoms, which act as possible adsorption centers of the inhibitor. This was corroborated because, in acidic environments, it had the highest E_HUMO_ value, the best chemical reactivity, the greatest tendency to transfer electrons and a greater facility of modifying its electronic configuration in the presence of carbon steel specimens according to its chemical hardness value. This explained why α-linolenic acid was more efficient in an acidic environment than in a neutral one.

## Figures and Tables

**Table 1:** Effect of α-linolenic acid concentration on the activation energy for 1018 carbon steel in 0.5 M H_2_SO_4_.

C_inh_ (ppm)	E_a_ (kJ mol^−1^)
0	33.41
100	60.18
200	63.42
400	83.47
600	83.64
800	93.66
1000	104.96

**Table 2:** Electrochemical parameters obtained from polarization curves.

C_inh_ (ppm)	E_corr_ (mV)	I_corr_ (mA/cm^2^)	β_a_ (mV/dec)	β_c_ (mV/dec)	I_pas_ (mA/cm^2^)	R_p_ (Ohm cm^2^)	I.E. (%)
0	−428	5.49	42	−88	0.30	18	-
100	−451	4.89	38	−140	0.29	75	24
200	−423	1.58	45	−160	0.08	106	75
400	−439	0.09	40	−165	0.16	540	96
600	−473	0.05	50	−190	0.25	594	97
800	−454	0.15	60	−270	0.16	184	96
1000	−475	0.23	50	−240	0.26	141	94

**Table 3:** Electrochemical parameters used to simulate the EIS results.

C_inh_ (ppm)	R_s_ (Ohm cm^2^)	R_ct_ (Ohm cm^2^)	CPE_dl_ (µF cm^2^)	R_f_ (Ohm cm^2^)	CPE_f_ (µF cm^2^)	I.E. (%)
0	4	28	7.99 × 10^−5^	-	-	-
100	7	140	4.47 × 10^−5^	15	8.47 × 10^−4^	51
200	8	420	2.57 × 10^−5^	86	4.57 × 10^−4^	78
400	8	1130	1.13 × 10^−5^	110	1.13 × 10^−4^	97
600	10	1960	7.08 × 10^−6^	110	6.38 × 10^−5^	98
800	11	120	4.27 × 10^−5^	50	3.27 × 10^−4^	78
1000	11	108	3.31 × 10^−5^	20	6.31 × 10^−4^	72

**Table 4:** Calculated quantum chemical parameters for the neutral α-linolenic acid molecule.

Chemical Properties	Values
E_HOMO_ (Hartree)	−0.23646
E_LUMO_ (Hartree)	−0.00920
E_HOMO_ (eV)	−6.43440
E_LUMO_ (eV)	−0.25034
GAP (eV)	6.1841
Electronegativity (eV)	3.1
Hardness (eV)	3.29
Ionization Potential (eV)	6.39
Dipole Moment	1.9342
Electrophilicity	1.46
Electron Affinity (eV)	−0.19

**Table 5:** Fukui functions, local softness, philicity, dual Fukui functions, dual local softness and dual local philicity for neutral α-linoleic acid molecule with Hirsfeld charges.

Atom	*f*_k_^+^	*f*_k_^−^	Δ*f*_k_	*S*_k_^+^	*S*_k_^−^	Δ*S*_k_	*ω*_k_^+^	*ω*_k_^−^	Δ*ω*_k_
3C	0.0586	0.0680	0.0094	0.0964	0.1119	0.0155	0.0855	0.0993	0.0138
4C	0.0475	0.0612	0.0137	0.0781	0.1006	0.0225	0.0693	0.0893	0.0199
7C	0.0915	0.0592	−0.0323	0.1505	0.0973	−0.0532	0.1336	0.0864	−0.0472
9C	0.0477	0.1083	0.0605	0.0785	0.1781	0.0996	0.0697	0.1581	0.0884
10C	0.0586	0.1133	0.0547	0.0964	0.1863	0.0899	0.0856	0.1653	0.0798
11C	0.0103	0.0210	0.0107	0.0169	0.0345	0.0176	0.0150	0.0306	0.0156
18C	0.0475	0.0002	−0.0473	0.0782	0.0004	−0.0778	0.0694	0.0003	−0.0691
19O	0.0428	0.0001	−0.0426	0.0703	0.0002	−0.0701	0.0624	0.0002	−0.0623
20O	0.0236	0.0004	−0.0232	0.0389	0.0006	−0.0382	0.0345	0.0006	−0.0339

**Table 6:** Calculated quantum chemical parameters for the protonated α-linolenic acid molecule.

Chemical Properties	Values
E_HOMO_ (Hartree)	−0.26663
E_LUMO_ (Hartree)	−0.12105
E_HOMO_ (eV)	−7.25537
E_LUMO_ (eV)	−3.29393
GAP (eV)	3.961
Electronegativity (eV)	5.30
Hardness (eV)	1.95
Ionization Potential (eV)	7.26
Dipole Moment	9.1693
Electrophilicity	7.20
Electron Affinity (eV)	3.35

**Table 7:** Fukui functions, local softness, local philicity, dual Fukui functions, dual local softness and dual local philicity for the protonated α-linoleic acid molecule with Hirsfeld charges.

Atom	*f*_k_^+^	*f*_k_^−^	Δ*f*_k_	*S*_k_^+^	*S*_k_^−^	Δ*S*_k_	*ω*_k_^+^	*ω*_k_^−^	Δ*ω*_k_
2C	0.0572	0.1610	0.1038	0.0940	0.2648	0.1708	0.0835	0.2350	0.1515
3C	0.0220	0.1777	0.1557	0.0362	0.2923	0.2561	0.0321	0.2594	0.2273
5C	0.1937	0.0133	−0.1804	0.3187	0.0219	−0.2968	0.2829	0.0195	−0.2634
7C	0.0720	0.0222	−0.0498	0.1185	0.0366	−0.0819	0.1052	0.0325	−0.0727
8C	0.0809	0.0175	−0.0634	0.1331	0.0288	−0.1043	0.1181	0.0256	−0.0925
18C	−0.0004	0.0432	0.0436	−0.0007	0.0710	0.0717	−0.0006	0.0630	0.0636
40H	0.0594	0.0121	−0.0473	0.0977	0.0199	−0.0779	0.0867	0.0176	−0.0691
44H	0.0206	0.0495	0.0290	0.0338	0.0815	0.0476	0.0300	0.0723	0.0423
45H	0.0156	0.0477	0.0321	0.0257	0.0785	0.0528	0.0228	0.0697	0.0469

## Data Availability

The data presented in this study are available on request from the corresponding author.
